# A Beta-Prototype Chatbot for Increasing Health Literacy of Patients With Decompensated Cirrhosis: Usability Study

**DOI:** 10.2196/42506

**Published:** 2023-08-15

**Authors:** Jessica Au, Caitlin Falloon, Ayngaran Ravi, Phil Ha, Suong Le

**Affiliations:** 1 School of Clinical Sciences Monash University Clayton Australia; 2 Department of Gastroenterology and Hepatology Monash Health Clayton Australia; 3 Monash Digital Therapeutics and Innovation Laboratory Monash University Clayton Australia

**Keywords:** chronic liver disease, chatbot, artificial intelligence, health literacy, acceptability

## Abstract

**Background:**

Health literacy is low among patients with chronic liver disease (CLD) and associated with poor health outcomes and increased health care use. Lucy LiverBot, an artificial intelligence chatbot was created by a multidisciplinary team at Monash Health, Australia, to improve health literacy and self-efficacy in patients with decompensated CLD.

**Objective:**

The aim of this study was to explore users’ experience with Lucy LiverBot using an unmoderated, in-person, qualitative test.

**Methods:**

Lucy LiverBot is a simple, low cost, and scalable digital intervention, which was at the beta prototype development phase at the time of usability testing. The concept and prototype development was realized in 2 phases: concept development and usability testing. We conducted a mixed methods study to assess usability of Lucy LiverBot as a tool for health literacy education among ambulatory and hospitalized patients with decompensated CLD at Monash Health. Patients were provided with free reign to interact with Lucy LiverBot on an iPad device under moderator observation. A 3-part survey (preuser, user, and postuser) was developed using the Unified Acceptance Theory Framework to capture the user experience.

**Results:**

There were 20 participants with a median age of 55.5 (IQR 46.0-60.5) years, 55% (n=11) of them were female, and 85% (n=17) of them were White. In total, 35% (n=7) of them reported having difficulty reading and understanding written medical information. Alcohol was the predominant etiology in 70% (n=14) of users. Participants actively engaged with Lucy LiverBot and identified it as a potential educational tool and device that could act as a social companion to improve well-being. In total, 25% (n=5) of them reported finding it difficult to learn about their health problems and 20% (n=4) of them found it difficult to find medical information they could trust. Qualitative interviews revealed the conversational nature of Lucy LiverBot was considered highly appealing with improvement in mental health and well-being reported as an unintended benefit of Lucy LiverBot. Patients who had been managing their liver cirrhosis for several years identified that they would be less likely to use Lucy LiverBot, but that it would have been more useful at the time of their diagnosis. Overall, Lucy LiverBot was perceived as a reliable and trustworthy source of information.

**Conclusions:**

Lucy LiverBot was well received and may be used to improve health literacy and address barriers to health care provision in patients with decompensated CLD. The study revealed important feedback that has been used to further optimize Lucy LiverBot. Further acceptability and validation studies are being undertaken to investigate whether Lucy LiverBot can improve clinical outcomes and health related quality of life in patients with decompensated CLD.

## Introduction

Chronic liver disease (CLD) is a major global public health burden and results in 2 million deaths annually [1,2]. Decompensated CLD is a significant contributor to patient morbidity and mortality and is defined as an acute deterioration in hepatic function resulting in jaundice, hepatic encephalopathy, ascites, hepatorenal syndrome, or spontaneous bacterial peritonitis [3,4]. In 2012, the direct health care costs associated with the treatment of liver disease was estimated at US $448 million in Australia [5] and US $32.5 billion in the United States [6]. Lost productivity costs in Australia were estimated at US $4.3 billion in 2012, mainly from lost lifetime earnings due to reduced life expectancies and lower employment participation [3]. The World Health Organization [7] defines health literacy as “the achievement of a level of knowledge, personal skills and confidence to take action to improve personal and community health by changing personal lifestyles and living conditions. Thus, health literacy means more than being able to read pamphlets and make appointments. By improving people’s access to health information, and their capacity to use it effectively, health literacy is critical to empowerment.”

Poor health literacy has been demonstrated in patients with CLD, which may contribute to the high morbidity, mortality, and economic burden experienced by this specific chronic disease cohort [8,9].

Adherence to chronic disease treatment regimes has also been associated with health literacy, with adherence rates being 14% higher in patients with higher levels of health literacy [10]. In patients with liver cirrhosis, simple educational interventions increased patient’s disease knowledge by 26% [11].

Furthermore, the low levels of health literacy combined with high unemployment rates act as significant barriers for such patients to navigate complex health care systems and communicate with clinicians [12,13]. Studies have demonstrated an association between education level and CLD mortality, and this association was magnified for those with alcohol-related etiology [14,15]. The epidemiology of CLD is shifting away from chronic viral hepatitis toward lifestyle related etiologies including alcohol abuse and metabolic syndrome. This highlights the need for targeted interventions which address health literacy to improve self-management by reducing alcohol consumption, and addressing obesity, malnutrition, and sarcopenia [3]. Hepatic encephalopathy also impacts patient’s ability to understand health information, as it impairs executive function, problem-solving, and attention [9].

There are limited studies reporting the true prevalence of poor health literacy, its etiology, and the identification and management of potentially modifiable or preventable risk factors for poorer health literacy in decompensated CLD. Liver cirrhosis is a multisystem disorder, which is difficult for both clinicians and patients to optimize according to guideline-based management. There are high expectations placed on patients and carers to manage complicated medication regimes, lactulose self-titration, fluid and salt restriction, and nutrition optimization in the community. It has not yet been demonstrated whether improved health literacy is associated with increased patient self-sufficiency in these domains of cirrhosis self-management. Patients with CLD also have significant carer requirements, which negatively impacts the mental, physical, and social well-being of patients and caregivers [16]. This burden is further amplified in patients with hepatic encephalopathy and cognitive dysfunction [17].

A novel strategy to improve chronic disease patient engagement and self-management are artificial intelligence (AI) “Chatbots.” Chatbots are an emerging health care technology used for basic diagnostic or monitoring purposes in ambulatory settings [18,19]. An AI chatbot has the ability to use natural language processing (NLP) to decipher human language in order to retrieve relevant data using conversational algorithms [20]. This interactive user interface, which is intended to simulate a bidirectional conversation with a clinician aims to increase patient engagement and reduce information overload [21,22]. AI chatbots can be deployed through an omnichannel strategy: web-based, Facebook messenger, and mobile apps [23].

Recent studies have shown high levels of acceptance of health specific chatbots by users and physicians [21,24]. In psychiatry, a discipline where chatbots are more prevalent, they are used to screen for mental health disorders and are also capable of delivering cognitive behavioral therapy [25,26]. A key limitation of existing health care chatbots is their lack of human emotion [27,28] and limited focus on education [29] when they could be leveraged as a tool to improve health literacy among patients with complex chronic conditions. Providing targeted information to improve health literacy digitally could also help bridge the communication gap between patient and clinicians, while increasing patient autonomy [30].

A liver specific “Chatbot” that promotes CLD health literacy through an interactive conversational interface has not been reported in the literature. Our study aims to investigate whether a novel AI chatbot is an acceptable tool to provide health information to patients with decompensated CLD.

## Methods

### Study Design

We conducted a prospective mixed method study to determine patient usability of “Lucy LiverBot,” an AI chatbot designed and built by a clinical multidisciplinary team (MDT) at Monash Health, Australia using a no code platform provided by software developers Andi Chatterton and Mark Chatterton from inGeniousAI, an industry partner.

### Lucy LiverBot

#### Overview

**Figure 1:**
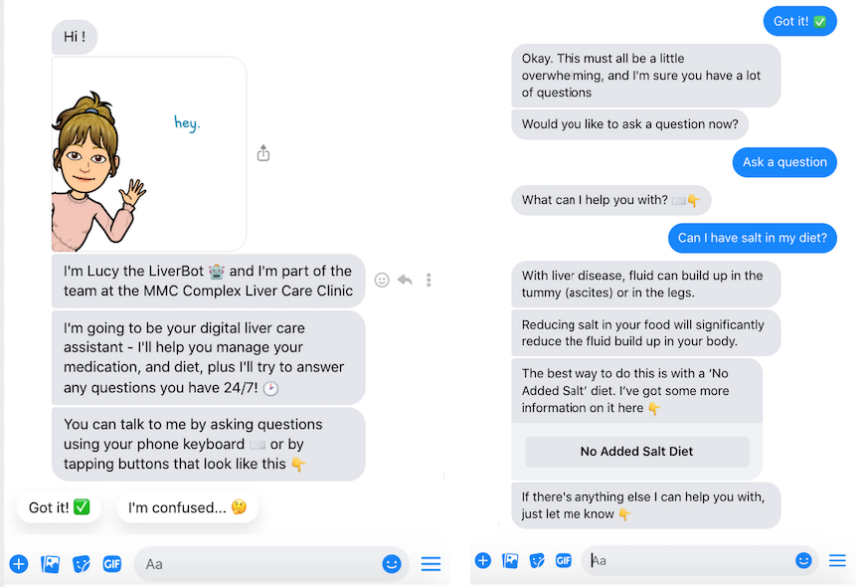
An example of a simple flow conversation with Lucy LiverBot which allows patients to type any questions they may have about chronic liver disease.

Lucy LiverBot is an AI chatbot developed by a MDT to deliver disease, medication, and nutrition-specific health information to patients with decompensated CLD (Figure 1). A key function of Lucy LiverBot is the emphasis on health literacy and education [8]. Information is presented to patients through conversational scripts, visuals, and videos in English.

#### Concept Development

Lucy LiverBot was developed by a MDT from Monash and Austin Health in 2019 comprising a Hepatologist, a Liver Nurse Consultant, a Liver Pharmacist, and a Liver Transplant Dietitian. Each member of the MDT team was responsible for identifying a list of 10 questions commonly posed by patients with CLD in their area of subject matter expertise. Each MDT member was then responsible for creating the answers to these questions and for the veracity of the content. In-depth interviews and small focus groups were also conducted with 10 patients attending the Complex Liver Care Clinic, Monash Health—an ambulatory care program for adults with decompensated cirrhosis to validate the questions to be answered by Lucy LiverBot, identify any missing questions, and confirm a patient need for the product.

The Liver Pharmacist was trained as a superuser, built the Lucy LiverBot, tested the NLP, and was the primary data custodian of the frequently asked questions bank. Technical support was provided by inGeniousAI to the Liver Pharmacist who built Lucy LiverBot on the inGeniousAI no code proprietary platform. This cross functional collaboration used an agile development approach, which involved iterative cycles of design, build, and testing with clinicians and patients. To use the chatbot, patients type in words or phrases into the text section. The chatbot then uses NLP to understand the intent of what the patient has typed and responds by extracting a scripted answer in our proprietary frequently asked questions library. Questions that cannot be answered by Lucy LiverBot are notified by email to the Liver Pharmacist who consults the relevant subject matter expert and then builds the response to this new question in the backend. Although all patients used written cues, the device also allows for voice-to-text recognition. Emojis were also used to reduce the amount of written text.

#### Usability Testing

We conducted the study from when standardized tools to assess user’s satisfaction with the experience of using chatbots were unavailable. We did not use other usability tools such as the System Usability Scale or the Usability Metric for User Experience, as these tools were not developed to consider the conversational aspects which relate to a user’s interaction with a chatbot. Instead, we used a mixed methods approach to gather preliminary insights into a patient’s experience with Lucy LiverBot. Each participant engaged in a 1-time only testing session consisting of (1) a preuser testing survey to determine patient demographics, their baseline confidence levels managing their own health, their understanding of CLD nutrition and possible barriers to optimal health; (2) a user-testing survey to determine patient satisfaction and to analyze whether the user interface is patient friendly; (3) a postuser testing survey to determine overall satisfaction with the app and its likely use in the management of CLD. All 3 surveys were developed using the Unified Acceptance Theory Framework [31].

The presurvey was developed by JA and PH and reviewed by SL, a consultant gastroenterologist and hepatologist. The presurvey was a validated questionnaire based on patient reported measures of treatment burden—the “Patient Experience with Treatment and Self-Management (PETS)” [32]. The user testing phase was made up of 2 sections: participant use of the chatbot and a subsequent survey. Lucy LiverBot was preloaded onto iPads which were provided to participants who could ask any nutrition-related questions for approximately 15 minutes. Within this time frame, Lucy LiverBot would guide participants through specific conversation flows depending on the key words used in their initiating question. The user testing survey questions were developed with input from inGeniousAI as their experience in chatbot design and deployment provided a valuable insight into the strategies required for successful user testing. The post user testing survey occurred immediately after the user testing survey and was developed to assess overall patient acceptance and usability of the app. The surveys were self-administered but with the moderator present, documenting additional feedback verbally provided by the patients throughout the testing session.

### Recruitment

Adult patients were recruited from liver clinics and inpatient wards at Monash Health, the second largest tertiary health care network in Australia. Monash Health provides 4.1 million episodes of care per year to a population of 1.5 million people. Given the intent to conduct a study, only 20 participants were recruited into this study. Study inclusion criteria were adults with decompensated CLD and capacity to provide informed consent. Clinical, laboratory, and imaging data were used to confirm patient CLD decompensation status. Decompensation was defined as per English as a second language criteria [33]. Participants were excluded from the study if they had greater than grade 1 hepatic encephalopathy at the time of consent, did not complete all components of the survey, or if they were unable to read, understand, or answer questions fluently in English.

### Statistical Analysis

Baseline clinical and disease demographics including current state of liver cirrhosis, decompensation complications, and total burden of hospital admissions in the past 12 months were extracted from the patient medical record. Summary data are presented as means (SD), proportions, or median (IQR) depending on the data distribution. All verbal patient feedback was documented and captured by the moderator verbatim. Anonymized transcripts were uploaded onto NVivo (Lumivero) for Windows (version 1.3; Microsoft Corp) for data management and coding. Qualitative data were reviewed by 2 independent assessors and 2 sets of key themes were identified. A third independent assessor then synthesized these results to produce a final set of key themes. Illustrative quotes were reported to support themes.

### Ethics Approval

Ethics approval (RES-19-461A) was granted by the Human Research Ethics Committee Monash Health and was carried out according to the National Statement on Ethical Conduct in Human Research (2018).

## Results

### Overview

The median age was 55.5 (IQR 46.0-60.5) years, with 55% (n=11) of them being female and 85% (n=17) of them being White (Table 1). The median BMI was 31.2 (IQR 22.6-36.55). Active alcohol consumption was cross referenced from both self-reporting and clinical documentation of alcohol being the confirmed etiology for CLD. No current alcohol intake was reported among 25% (n=5) of participants, 50% (n=10) of them were still current drinkers and 25% (n=5) of them had quit drinking alcohol.

**Table 1:** Baseline demographics (n=20).

Characteristics		Value
Age (years), median (IQR)		55.5 (46.0-60.5)
**Sex, n (%)**		
	Male	9 (45)
BMI, median (IQR)		31.2 (22.6-36.55)
**Ethnicity, n (%)**		
	White	17 (85)
**Smoking status, n (%)**		
	Smoker or ex-smoker	11 (55)
**Alcohol intake, n (%)**		
	Previous or current alcohol intake	15 (75)
**English fluency, n (%)**		
	Fluent	18 (90)
**Highest level of education, n (%)**		
	Less than high school	12 (60)
	High school graduate	8 (30)
	Tertiary	2 (10)
**Do you have difficulty reading and understanding medical information? n (%)**		
	Never	13 (65)
	Sometimes	5 (25)
	Usually	2 (10)
**Employment status, n (%)**		
	Unemployed	19 (95)
**Carer, n (%)**		
	Yes	14 (70)
**Owns a device, n (%)**		
	Yes	18 (90)
**Cause of cirrhosis, n (%)**		
	Alcohol	14 (70)
	Viral	3 (15)
	Other	3 (15)
**Time since diagnosis of cirrhosis, n (%)**		
	Unsure	7 (35)
	<6 months	2 (10)
	6 months to 2 years	4 (20)
	2-4 years	4 (15)
	>4 years	3 (15)

In total, 35% (n=7) of participants reported having difficulty reading and understanding written medical information, despite 90% (n=18) of participants being fluent in English. A large proportion had not completed high school (n=12, 60%) and were unemployed (n=19, 95%); a majority (n=14, 70%) required a carer. The primary cause of liver cirrhosis in this patient group was alcohol (n=14, 70%), followed by viral (n=3, 15%) and other (n=3, 15%).

Of the 20 participants, 25% (n=5) of participants found it difficult to learn about their health problems and 20% (n=4) of them found it difficult to find medical information they could trust (Table 2). Although 65% (n=13) of them found it easy to understand advice provided directly by their health care providers, and 25% (n=5) of them found it difficult to understand. In addition, 20% (n=4) of them found it difficult to find information on what foods they should eat to stay healthy and 45% (n=9) of them reported issues monitoring their eating and drinking habits. In addition, 70% (n=14) of them were bothered by feeling dependent on others for health care needs, with 35% (n=7) of them bothered when family or friends reminded them to do things for their health. Regarding emotional well-being, 50% (n=10) of them felt preoccupied by their self-care, with 55% (n=11) of them depressed about their CLD. A large proportion felt worn out by self-care (n=14, 70%) and were frustrated (n=15, 75%) with their health situation (Table 2).

**Table 2:** Difficulties experienced by patients in the self-management of decompensated cirrhosis.

Survey questions			Participants, n (%)
**Understanding medical information**			
	**How easy** **or** **difficult has it been to learn about your health problems?**		
		Easy	12 (60)
		Neither easy or difficult	3 (15)
		Difficult	5 (25)
	**How easy** **or** **difficult has it been to learn what foods you should eat to stay healthy?**		
		Easy	14 (70)
		Neither easy or difficult	2 (10)
		Difficult	4 (20)
	**How easy** **or** **difficult has it been to find sources of medical information that you trust?**		
		Easy	15 (75)
		Neither easy or difficult	1 (5)
		Difficult	4 (20)
	**How easy** **or** **difficult has it been to understand advice from different health care providers?**		
		Easy	13 (65)
		Neither easy or difficult	2 (10)
		Difficult	5 (25)
**Monitoring health behaviors**			
	**How much of a problem has it been for you to monitor your health behaviors, for example, exercise, diet and medication adherence?**		
		A little	12 (60)
		Somewhat	3 (15)
		Quite a bit	4 (20)
		Not applicable	1 (5)
	**How bothered have you been by feeling dependent on others for your health care needs?**		
		A little	5 (25)
		Somewhat	7 (35)
		Quite a bit	6 (30)
		Not applicable	2 (10)
	**How bothered have you been by others reminding you to do things for your health, for example, take medications, eat healthy, schedule appointments?**		
		A little	14 (70)
		Somewhat	1 (5)
		Quite a bit	4 (20)
		Not applicable	1 (5)
**Managing emotional well-being**			
	**How often did your self-care make you feel preoccupied?**		
		Rarely	8 (40)
		Sometimes	8 (40)
		Often	2 (10)
		Not applicable	2 (10)
	**How often did your self-care make you feel depressed?**		
		Rarely	8 (40)
		Sometimes	9 (45)
		Often	2 (10)
		Not applicable	1 (5)
	**How often did your self-care make you feel worn out?**		
		Rarely	6 (30)
		Sometimes	6 (30)
		Often	8 (40)
		Not applicable	0 (0)
	**How often did your self-care make you feel frustrated?**		
		Rarely	5 (25)
		Sometimes	8 (40)
		Often	7 (35)
		Not applicable	0 (0)
**Problems with multidisciplinary communication**			
	**I have problems with different health care providers not communicating with each other about my medical care?**		
		Agree	4 (20)
		Neither agree or disagree	4 (20)
		Disagree	10 (50)
		Not applicable	2 (10)

### Qualitative Results

#### Mental Health and Well-Being

Several participants identified improvement in mental health and well-being as an unintended benefit of Lucy LiverBot. Beyond providing disease specific information, the conversational nature of the chatbot appealed to many as it provided a well-received reminder to maintain habits conducive to their well-being and health habits.

Maintaining wellbeing.

Keep checking up on you.

It was 1 participant who suggested additions that could be incorporated into the chatbot to specifically focus on the mental health of users.

Something for anxiety and depression could help, particularly being able to write a journal.

Lucy LiverBot was overtly identified as a potential “companion” by patients with CLD by providing a sense of social connection for patients who are socially isolated.

You can talk like you are talking to somebody else like a friend.

#### Timing of Chatbot Implementation

A common theme that emerged from participants was that the use of Lucy LiverBot may depend on the timing of its implementation in the patient’s disease progress. Patients who had been managing their liver cirrhosis for longer periods of time identified that they would be less likely to use Lucy LiverBot at later stages of CLD, but that it would have been useful at the time of their diagnosis.

Telling stuff I already know so not that useful.

Brilliant, can help many people, for young people.

#### Reliable Source of Medical Information

Lucy LiverBot was generally perceived as a reliable and trustworthy source of information as it was produced by medical professionals in the field of CLD. Participants recognized their potential to provide a trusted reference for nutritional information, rather than resorting to the internet.

Having information that is not conflicting.

Very informative. Would explain everything if I didn't know anything about cirrhosis.

It's quick and very simple to use. I like how I can ask questions as soon as they arise rather than wait for an appointment or google world wide.

## Discussion

### Principal Findings

There is a clinical urgency for cost-effective and scalable interventions that address the poor health literacy of patients with CLD [34] in order to improve patient engagement and self-management of this complex condition [35,36]. Lucy LiverBot was well received by participants and the results suggest that it could provide targeted CLD information via an engaging channel. Participants were actively engaged while using Lucy LiverBot throughout the in-depth user testing process, which took approximately 1 hour. We were also able to capture users from a variety of age groups and at different stages of their disease process which allowed us to determine at what stage of CLD Lucy LiverBot would be most useful. In addition, our extensive testing process ensured that all available chat flows were tested and NLP continued to improve with each consecutive patient.

Our results highlighted key barriers faced by patients with CLD which have the potential to impact their health outcomes—understanding health information, monitoring health behaviors, managing emotional well-being, and multidisciplinary communication. Lucy LiverBot has been specifically designed to assist patients with the understanding of health information and the monitoring of their health behaviors. It is also hoped that a centralized digital device designed by the MDT will help bridge the communication gap between patients and clinicians.

Many participants identified that the conversational tone and companion-like nature of the chatbot was one of its key strengths. Lucy LiverBot’s ability to engage with users provided a social platform for them to ask concerns and may have the potential to extend its disease specific content to directly address mental well-being and provide a sense of social connection. By addressing these identified barriers, Lucy LiverBot has the potential to fill a gap in the provision of health care to this group of complex chronic disease patients. Further validation studies are required to determine whether Lucy LiverBot as an intervention would prevent clinical outcomes such as readmission related to decompensated CLD.

It would be important to continue monitoring the performance of Lucy LiverBot after its launch to identify any errors in NLP so that necessary adjustments can be made. The NLP feature in Lucy LiverBot is basic and further advancements in this technology will be required to improve future iterations capable of providing an even more engaging user experience. The user testing allowed us to gauge how patients were most likely to phrase questions which allowed us to alter recognized terms. This was evident as Lucy LiverBot was unable to recognize some patient questions during the user testing stage if they were not worded in a similar manner to the initial input options. Unfortunately, the chatbot is currently only available in English, which limits its generalizability and scalability for participants from Culturally and Linguistically Diverse communities. This precluded some patients from participating in this study, however this is a technical limitation of NLP in general, rather than of Lucy LiverBot specifically. Ideally, future versions of Lucy LiverBot will be available in multiple languages.

The study was limited by a paucity of research on health chatbots, which made it difficult to determine the sample size required to adequately power the study and the ideal study design to assess patient usability. Our small study population allowed preliminary information to be obtained regarding the usability of Lucy LiverBot and its potential to act as an educational tool for patients with CLD. However, future studies with larger cohorts of patients will be required to definitively demonstrate Lucy LiverBot’s ability to improve health literacy and health outcomes. It is likely that solutions such as Lucy LiverBot will require frequent cycles of iteration and user testing to become maximally effective. This will lend more insight into what features are most beneficial within Lucy LiverBot and whether patients will be committed to using it for an extended period of their own volition beyond a study context. There was a potential selection bias as participants who agreed to join the study may be more motivated to improve their health and more likely to engage with Lucy LiverBot. In addition, response bias may have played a role in the study as participants completed the survey while investigators were in the room for technical support. This may have influenced patients to select answers that they believed were more acceptable. To remove any potential responder bias due to a perceived impact on their care, participants were assured that the results from the study would not be viewed by their treating team. We also trained final year medical students to conduct the testing rather than physicians to reduce the perceived power imbalance between participants and interviewers. The study also did not assess the stage of hepatic encephalopathy in participants. In future studies, we plan to assess this both at baseline and longitudinally to further delineate the effect of hepatic encephalopathy on a patient’s ability to remember health specific information.

The efficacy of novel digital health interventions such as Lucy LiverBot, which lack a formal evaluation framework akin to pharmacotherapy and device trials, would benefit from a multidisciplinary evaluation strategy tailored to the specific study end point. We conducted the study from when standardized tools to assess user’s satisfaction with the experience of using chatbots were unavailable. If we were to repeat the study again we would leverage new tools such as the Chatbot Usability Scale. An assessment of human computer interactions will also be required to determine the real-world patient usage patterns of Lucy LiverBot. A randomized controlled trial would be the ideal format in determining whether Lucy LiverBot is effective in improving health literacy and reducing hospital readmission. However, as ambulatory medical care models become increasingly multidisciplinary, it may become difficult to delineate which arms of the multimodal health care model are responsible for changes in clinical outcome. For example, if an improvement in admissions for hepatic encephalopathy were to be observed, this could be attributed to the increased communication with clinical staff through chatbot alerts, the health education provided by the chatbot, or perhaps an improvement in other indices such as nutrition and adherence. Further prospective studies based on the principles of implementation science are warranted to assess the benefits that Lucy LiverBot may provide to clinical end points such as decompensation rate, morbidity, quality of life, and clinic attendance. A longitudinal component of the study should be established whereby participants are tracked throughout their disease progression and compared to those who did not use a health chatbot to determine whether Lucy LiverBot prevented hospital readmissions and led to improved patient outcomes. Such studies will need to perform costings analyses and assess long-term patient participation, and adherence to digital health care models.

### Conclusions

Our study identified barriers to health care provision and found that Lucy LiverBot was well received by patients with decompensated chronic liver disease. Lucy LiverBot can specifically address these barriers and be introduced as a potential educational intervention to address the impact of poor health literacy on disease outcomes and a health related quality of life. Further validation studies are required to demonstrate the potential for Lucy LiverBot to improve patient engagement and self-management and its use as an engagement tool with multidisciplinary teams.

## Abbreviations

AI: artificial intelligence
CLD: chronic liver disease
MDT: multidisciplinary team
NLP: natural language processing
PETS: Patient Experience with Treatment and Self-Management
